# Renocardiac syndromes: physiopathology and treatment stratagems

**DOI:** 10.1186/s40697-015-0075-4

**Published:** 2015-10-16

**Authors:** J. G. Kingma, D. Simard, J. R. Rouleau

**Affiliations:** Faculté de Médecine, Pavillon Ferdinand-Vandry, 1050, Ave de la Médecine, Université Laval, Québec, G1V 0A6 Canada; Centre de Recherche, Institut de Cardiologie et Pneumologie de Québec, 2725, Chemin Sainte-Foy, Québec, G1V 4G5 Canada

## Abstract

**Purpose of review:**

Bidirectional inter-organ interactions are essential for normal functioning of the human body; however, they may also promote adverse conditions in remote organs. This review provides a narrative summary of the epidemiology, physiopathological mechanisms and clinical management of patients with combined renal and cardiac disease (recently classified as type 3 and 4 cardiorenal syndrome). Findings are also discussed within the context of basic research in animal models with similar comorbidities.

**Sources of information:**

Pertinent published articles were identified by literature search of PubMed, MEDLINE and Google Scholar. Additional data from studies in the author’s laboratory were also consulted.

**Findings:**

The prevalence of renocardiac syndrome throughout the world is increasing in part due to an aging population and to other risk factors including hypertension, diabetes and dyslipidemia. Pathogenesis of this disorder involves multiple bidirectional interactions between the kidneys and heart; however, participation of other organs cannot be excluded. Our own work supports the hypothesis that the uremic milieu, caused by kidney dysfunction, produces major alterations in vasoregulatory control particularly at the level of the microvasculature that results in impaired oxygen delivery and blood perfusion.

**Limitations:**

Recent clinical literature is replete with articles discussing the necessity to clearly define or characterize what constitutes cardiorenal syndrome in order to improve clinical management of affected patients. Patients are treated after onset of symptoms with limited available information regarding etiology. While understanding of mechanisms involved in pathogenesis of inter-organ crosstalk remains a challenging objective, basic research data remains limited partly because of the lack of animal models.

**Implications:**

Preservation of microvascular integrity may be the most critical factor to limit progression of multi-organ disorders including renocardiac syndrome. More fundamental studies are needed to help elucidate physiopathological mechanisms and for development of treatments to improve clinical outcomes.

## Abrégé

**Objectifs de la révision:**

Les interactions bidirectionnelles entre organes adjacents sont essentielles au bon fonctionnement du corps humain mais sont aussi susceptibles de provoquer des conditions adverses sur des organes plus éloignés. Cette revue offre un compte rendu sommaire de l’épidémiologie, des mécanismes physiopathologiques et du traitement clinique des patients atteints à la fois d’insuffisance rénale et de cardiopathie, ou tel que récemment désignés, atteints du syndrome cardiorénal de type 3 ou de type 4. La revue examine également des résultats obtenus en recherche fondamentale en utilisant des modèles animaux présentant des cas similaires de comorbidité.

**Sources:**

Les articles pertinents ont été répertoriés à la suite d’une recherche dans la littérature sur PubMed, MEDLINE et « Google Scholar ». Des données complémentaires provenant  d’études du laboratoire de recherche de l'auteur ont aussi été consultées.

**Constatations:**

Le vieillissement de la population en plus de facteurs de risque incluant l’hypertension, le diabète et la dyslipidémie augmente en partie la prévalence du syndrome cardiorénal à travers le monde. La pathogenèse de ce désordre implique de multiples interactions bidirectionnelles entre le cœur et les reins; cependant, la participation d’organes périphériques n’est tout de même pas à exclure. Nos travaux soutiennent l’hypothèse selon laquelle l’environnement urémique résultant de la dysfonction rénale serait responsable d’altérations majeures dans la régulation de la pression, particulièrement au niveau des microvaisseaux. En résultent une perfusion sanguine altérée et une distribution insuffisante d’oxygène vers les organes.

**Limites de l’étude:**

La littérature clinique récente comporte de nombreux articles traitant de la nécessité d’identifier et de caractériser de façon plus élaborée les causes du syndrome cardiorénal dans la perspective d’améliorer le traitement clinique des patients qui en sont atteints. Par contre, puisqu’il existe encore très peu d’informations sur l’étiologie du syndrome cardiorénal, les patients ne sont pris en charge qu’après son apparition. Qui plus est, la compréhension des mécanismes impliqués dans la pathogenèse résultant des interactions entre organes demeure un objectif difficile à atteindre, en partie parce que la recherche fondamentale est limitée étant donné la rareté des modèles animaux pour cette pathologie.

**Conséquences:**

À la lumière des données disponibles à ce jour, il apparait que la préservation de l’intégrité du système vasculaire, particulièrement au niveau des microvaisseaux, est un facteur-clé pour restreindre le développement de désordres impliquant plusieurs organes tel le syndrome cardiorénal. Davantage d’études en recherche fondamentale sont requises pour faire la lumière sur les mécanismes physiopathologiques de ce syndrome et développer des traitements efficaces pour en améliorer les résultats cliniques.

## What was known before

Co-existence of kidney and cardiac disorders is increasingly prevalent throughout the world. A major consequence of failing kidneys is the stimulation of metabolic and humoral pathways that cause injury to remote organs; a similar scenario occurs with disorders of other organs such as the heart and liver. Mechanisms responsible for disease progression, regardless of the organ initially affected, are complex; understanding the mechanisms and pathways involved in, or responsible for inter-organ crosstalk, is a growing area of research interest. Clinical management of cardiorenal syndrome patients is particularly complex due to the involvement of multiple organs and the difficulty in targeting specific symptoms.

## What this adds

Herein, we review recent advances concerning physiopathology, therapeutic interventions and complications of renocardiac syndrome. Recent data, including our own, from animal models are discussed here; primary focus is on the impact of acute kidney injury on systemic hemodynamics, blood flow regulation and endothelial function. Novel therapies that target the microcirculation could benefit renocardiac syndrome patients and improve mortality.

## Introduction

Evaluation of underlying causes and physiopathological mechanisms responsible for kidney related disorders represents a significant challenge. Kidney and heart disease often co-exist; the heart is highly dependent on regulation of salt and water content by the kidneys that directly depend on blood flow and pressure generated by the heart. Functional deterioration of either organ initiates a vicious spiral of events that leads to multi-organ failure. Although prevalence of renal dysfunction in patients with heart disease is well known [1, 2] it remains unclear whether kidney failure is a passive response to failing cardiac performance. The co-existence of cardiac and renal pathologies in the same patient, referred to as cardiorenal syndrome (CRS) since 2004, is the subject of many contemporary studies. Furthermore, the concept of organ-to-organ crosstalk and the development of multi-organ dysfunction is more widely accepted. Clinical classification of CRS (cf. Table 1) is based on primary organ dysfunction; classification criteria have recently been reviewed by the Acute Dialysis Quality Initiative (ADQI) Working Group [3–6].

**Table 1:** Cardiorenal syndrome classification

Type 1 (acute cardio-renal syndrome)	Abrupt deterioration of cardiac function that results in acute kidney injury (AKI)
Type 2 (chronic cardio-renal syndrome)	Chronic abnormalities of cardiac function leading to progressive chronic kidney disease (CKD)
Type 3 (acute reno-cardiac syndrome)	Abrupt and primary worsening of kidney function that initiates acute cardiac dysfunction
Type 4 (chronic reno-cardiac syndrome)	CKD that promotes reduction of cardiac function
Type 5 (secondary CRS)	Systemic disorders that impair both cardiac and renal function

Herein, we provide a narrative review of clinical and basic science literature on renocardiac syndrome (type 3 and type 4 CRS) with regard to epidemiology, pathogenesis and clinical interventions designed to improve outcomes. Clinical and basic science reports were searched using MEDLINE and PubMed with the keywords reno-cardiac syndrome, kidney disease, heart disease and combinations thereof.

Type 3 CRS is usually triggered by an episode of acute kidney injury (AKI); nephrons are particularly sensitive to ischemia and blood borne toxins. AKI is often superimposed on chronic renal disease and could be a necessary precursor of end-stage renal disease. Elucidation of mechanisms remains difficult due to the complex interplay between chronic and acute kidney disease phenotypes [7]. Acute worsening of kidney function ultimately produces cardiac dysfunction (i.e. acute decompensated heart failure, acute myocardial infarction and arrhythmias) [8]. The overall incidence of AKI in the general population appears to be increasing [9] based on Risk, Injury, Failure, Loss, End-stage kidney disease classification (RIFLE)/Acute kidney injury network (AKIN) criteria [10] that use change in serum creatinine and urinary output as primary markers of kidney dysfunction.

Type 4 CRS, on the other hand, involves chronic abnormalities of renal function due in part to aging, diabetes, hypertension and dyslipidemia that progresses to multi-organ disease [11] possibly due to toxic effects of elevated uremia levels. Cardiovascular disease is highly prevalent in these patients and accounts for the majority of cardiac-related deaths (secondary to ischemia) [12].

## Epidemiology and risk factors

In the UK, The National Confidential Enquiry into Patient Outcome and Death (http://www.ncepod.org.uk) survey, published in 2009, discovered a systematic failure by hospital staff to recognize complications of AKI which ultimately resulted in poor clinical outcomes. The report also underscored the importance of preventing all-cause early transient malfunction of the kidneys due to irreversible structural damage. Even though almost 20 million American adults are known to be affected by some form of kidney disease [13] the proportion with Type 3 CRS (consequent to AKI) is not documented. Common risk factors responsible for acute renocardiac syndrome are summarized in Table 2. Cardiovascular related mortality is significantly elevated in patients with AKI [14, 15]. A prospective Spanish multicenter study almost a decade ago examined the relation between acute kidney failure and multi-organ failure and reported a significant increase in mortality (~30 %) in relation to the number of failed organs; that study was performed using intensive care unit and non-intensive care unit patients [16]. Surgery patients are also a high risk group for AKI due to the potential for marked renal hypoperfusion; [17] patients undergoing coronary artery bypass grafting with minor increases in post-operative serum creatinine had a higher occurrence of myocardial infarction and a 3-fold rise in long term risk of end-stage renal disease [18, 19]. Iodinated radiographic contrast media, commonly used for various clinical applications in patients with comorbidities, can also elicit significant kidney injury. Additional risk factors recently suggested to contribute to development of AKI-related pathology include body mass index, [20] proteinuria [21, 22] and microalbuminuria [23].

**Table 2:** Risk factors for AKI

Renal artery stenosis (ischemia-reperfusion injury)
Myocardial infarction
Surgical interventions (including anesthesia)
Trauma
Intrinsic/extrinsic ureteral obstruction
Dehydration
Infection (gastroenteritis, etc.)
Drug-related complications (pharmacologic toxicity, drug-abuse, etc.)

Clinical evaluation of Type 4 CRS is more obvious; responsible for almost 50 % of deaths in all age groups of CKD patients [24, 25]. Indeed, it has been suggested that CKD (see Table 3 for risk factors) be included on the listing of criteria for patients with high risk of coronary events [26, 27]. Defining the epidemiology of Type 4 CRS is problematic and clinical diagnosis of these patients is difficult due to variants in: 1- populations-at-risk, 2- clinical outcomes evaluated, 3- timeframes to determine study end-points and 4- definitions for CKD, cardiac disease and mortality [28]. However, higher hazard ratios for cardiovascular events and all-cause mortality in relation to decreases in glomerular filtration rate have been reported [29, 30]. A joint study by the United States Renal Data System and the National Registry of Myocardial Infarction reported a lower likelihood of chest pain in advanced CKD compared to non-CKD patients [31]. Finally, patients with CKD are less likely to receive evidence-based therapies because of their atypical clinical presentation profiles [32–34].

**Table 3:** Possible risk factors for CKD

AKI (all cause)
Hypertension, cardiovascular and hepatic disease
Diabetes
Age, gender, race
Obesity, smoking

## Mechanisms

Studies of the physiopathological evolution of kidney injury, either acute or chronic, in humans are rare possibly because of the common misconception that tubular regeneration occurs in patients after AKI [35–38]. This is based primarily on evidence of normalisation of serum creatinine levels. Potential mechanisms for Type 3 and 4 CRS are categorized on the basis of hemodynamic or non-hemodynamic criteria [5].

### Hemodynamic factors

Cardio-renal interactions are generally explained using extracellular fluid volume homeostasis and blood pressure control criteria [39]. Consequences of heart failure including reduced cardiac output and blood pressure stimulate both the sympathetic nervous and renin-angiotensin systems which results in volume expansion; [40, 41] the latter allows restoration of renal perfusion. Data for kidney hemodynamics and segmental sodium handling are limited for patients with combined heart and renal failure. However, bi-directional coupling between dysfunctional heart and kidneys induces sodium and water retention that ultimately exacerbates heart failure by affecting arterial pressure (lower) and renal venous pressure (higher). Treatment success rates in patients presenting with heart and kidney failure is mitigated. Additional data on the role of hemodynamic factors in progression of acute and chronic renocardiac syndrome can be obtained in animal models where the relation between renal venous pressure, renal blood flow, [42] intra-tubular pressure [43, 44] and glomerular filtration rate [45] has been established.

**Fig. 1:**
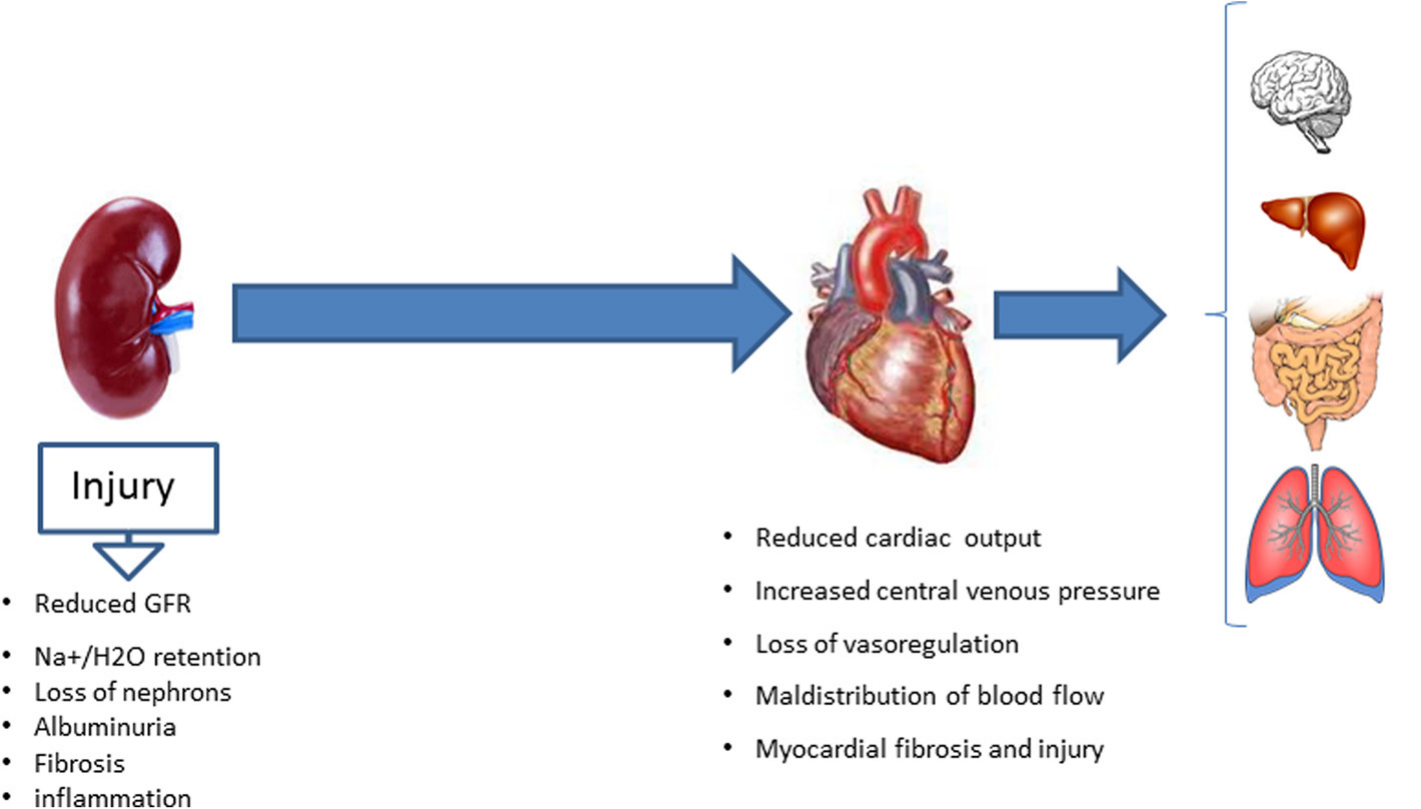
Schematic of pathways for kidney injury leading to heart and multi-organ failure

Progressive kidney dysfunction associated with chronic kidney disease, without either pharmacologic or non-pharmacologic intervention, ultimately results in multiple organ failure. Acid–base and electrolyte imbalance, fluid overload, atrial distension, hematologic dysfunction and diminished capacity to eliminate drugs all contribute. Physiopathologic mechanisms responsible for communications between kidney injury and cardiac dysfunction remain to be established; however, reduced cardiac performance ultimately limits blood perfusion of all organs including the kidneys and thereby contributes to renal injury. AKI affects the heart either 1- directly or 2- by limiting remote organ function which then indirectly influences cardiac function (cf. Fig. 1).

### Non-hemodynamic factors

In addition to the proposed hemodynamic factors, various cardiorenal connectors may activate endogenous systems after AKI and contribute to progression of symptoms. These include, but are not limited to, the sympathetic nervous, renin-angiotensin aldosterone and coagulation systems, [46] inflammation, oxidative stress and nitric oxide equilibrium.

For type 3 CRS, AKI (due to acute arterial ischemia-reperfusion injury, or other causes) produces rapid and significant functional changes in the heart characterized by LV dilatation and alterations of various functional parameters including LV relaxation time, fractional shortening and end-systolic and end-diastolic fractional shortening. Cardiocyte apoptosis has been suggested to play a role in promoting these changes along with stimulation of inflammatory mediators. Ischemia initiates a cascade of inflammation that is crucial to organ repair and if unchecked, eventual deterioration of organ function. In rodent models of acute and chronic kidney disease, the role of inflammation is predominant as evidenced by greater secretion of pro-inflammatory cytokines and infiltration of inflammatory cell types [47, 48]. The neuroendocrine system also plays an important role in physiopathology of type 3 CRS; complex pathways are activated after onset of AKI resulting in activation of the systemic nervous and renin-angiotensin systems. While activation of the systemic nervous system initially protects cardiac output it also appears to stimulate apoptosis, [49] neointimal formation and affects immune system function [8]. In addition, activation of the renin angiotensin system stimulates renin secretion by the kidneys; it also leads to dysregulation of extracellular fluid volume and vasoconstriction which can exacerbate the effects of ischemia by limiting adequate oxygen delivery.

Mechanisms involved in type 4 CRS are multi-faceted and invariably shared by different organs because of vascular disease and endothelial dysfunction as well as the cumulative toxic influences of uremia. A plethora of risk factors in these patients contributes to progression of cardiac and kidney failure; unique risks are also associated with dialysis procedures particularly in patients with end-stage renal disease [50]. A recent review by House provides an exclusive summary of potential mechanisms [24]. The role of the uremic milieu in development of multi-organ dysfunction still needs to be appraised; specific uremic toxins (guanidines, phenols, parathyroid hormone, proinflammatory cytokines, etc.), or combinations thereof, could directly cause metabolic and physiologic derangements and contribute to progression of the disease phenotype. In patients with congestive heart failure and progressive renal insufficiency, pressure and volume overload result in augmented cardiac work and compensatory hypertrophy (in part due to cardiac and renal fibrosis). Under these conditions oxygen delivery to enlarged myocytes is compromised due to vascular remodeling at the level of the microvasculature; this focal underperfusion or maldistribution of blood aggravates cellular injury.

**Fig. 2:**
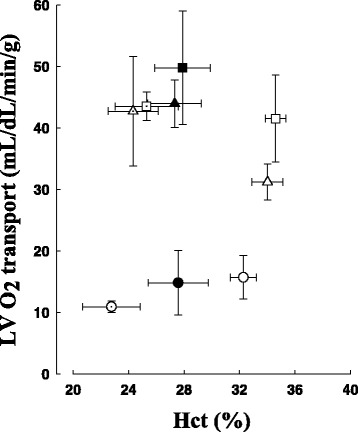
Change in LV oxygen transport versus hematocrit (Hct) for control dogs (*n* = 5, *open circle*) at baseline, dobutamine-5 (5 μg/Kg/min; *open triangle*) and dobutamine-10 (10 μg/Kg/min; *open square*). Stage 1 CKD (*n* = 5, closed symbols and stage 2 CKD (*n* = 5, *open dotted symbols*) are shown. In control dogs, LV oxygen transport increased in direct relation to intravenous dobutamine levels. In dogs with Stage 1 or 2 CKD, maximal LV oxygen transport was achieved at the lower dose of intravenous dobutamine compared to controls. Data shown are means ± 1SD

Using a two-stage subtotal nephrectomy uremia model (AKI by permanent occlusion of renal artery branches that produces type 3 CRS) we have been able to provide evidence for significant perfusion abnormalities across the ventricular wall in relation to severity of kidney dysfunction (assessed by serum creatinine) [51]. In normal animals myocardial blood flow increases in a dose-dependent fashion during dobutamine challenge (i.e. increased cardiac work); however, in uremic dogs even low-dose dobutamine maximally increased myocardial blood flow and oxygen transport (cf. Fig. 2). On the basis of these findings, we suggested the possibility of an increased risk of adverse coronary events due to the loss of transmural autoregulation and potential for maldistribution of myocardial perfusion. Renal autoregulation has also been shown to be significantly impaired during CKD; [52] this would exacerbate injury due to limited perfusion of blood. With more severe AKI we reported a significant rightward shift of the coronary perfusion pressure-blood flow relation and markedly blunted vessel reactivity to endothelium dependent/independent agonists; [53] these pre-clinical findings support the hypothesis that increased levels of uremic toxins can directly influence vasoregulation and endothelial function and thereby organ perfusion. The incidence of mortality was also markedly higher in dogs with elevated serum creatinine and blood urea nitrogen. Wang and Bao recently reported a significant correlation between serum uremia and endothelial dysfunction in rodents with early kidney disease [54]. We believe that the relevance of these findings to organ dysfunction merits future investigation as endothelial dysfunction, vascular calcification, and accelerated systemic inflammation all contribute to increased vascular stiffness and alteration of arterial pulse pressure and myocardial perfusion in patients with end-stage renal disease [55, 56].

Continued investigation to determine the physiopathological mechanisms involved in development of renal disease after AKI will require a multifaceted and bidirectional approach. Identification of risk factors involved in early kidney injury might be the most logical approach to prevent and delay adverse outcomes; [57] as stated earlier, vascular remodeling in the presence of uremic toxins increases oxidative stress, inflammation and lipid metabolism that exacerbates endothelial dysfunction. Thus, prevention of early microvascular dysfunction may be fundamental to limiting adverse effects of progressive kidney and heart disorders.

## Treatment strategies

Chuasuwan and Kellum recently reviewed different treatment strategies specific to the kidneys and heart based on RIFLE and AKIN criteria [8] that establish different severity levels for AKI to enable prediction of outcomes in affected patients. In high risk patients, potentially nephrotoxic drugs must be avoided and efforts should be directed to maintaining arterial pressure and preventing volume overload. Three stages (risk, injury, failure) of AKI were proposed. For stage 1 AKI patients, kidney function should be closely monitored (i.e. using non-invasive diagnostic evaluations). Stage 2 AKI presents particular challenges due to elevated risk of mortality; fluid, electrolyte and acid–base homeostasis is of particular concern. Stage 3 AKI is the most severe and life-threatening and often requires extracorporeal kidney support or renal replacement. Treatment strategies for the heart in type 3 CRS pose a particular challenge; prevention of LV volume overload is fundamental to limit the potential for worsening cardiac and renal function. Use of diuretics to improve clinical symptoms in heart failure patients is the *status quo*; however, evidence of a mortality benefit in patients with AKI remains controversial [58–60]. Indeed, use of diuretics for AKI is contra-indicated except for management of volume overload [61, 62]. Clinical outcomes are also improved using ultrafiltration and hemofiltration to reduce volume overload in patients that are refractory to diuretics.

Type 4 CRS patient management requires a multidisciplinary approach because of the bidirectional, multifaceted physiopathology of this syndrome; treatment strategies are mostly targeted to risk factors such as anemia, hypertension and malnutrition [63, 64]. However, for the most part , no specific treatment provides unequivocal benefit since targeted risk factors comprise only a small fraction of the physiopathological puzzle. As such, a combination therapy approach is probably needed to limit the devastating effects of this syndrome.

While there is no consensus for pharmacological management of Type 3 or 4 CRS patients, there is general agreement that more evidence-based clinical studies are necessary. Numerous inotropes and vasodilators including neurohormonal antagonists and diuretics have been evaluated on the basis of their ability to increase urine output and glomerular filtration and lower serum creatinine (see recent review by Kim [65]). Modest improvement of kidney function and survival (OPTIME-HF [66]) has been reported with milrinone in acute decompensated heart failure patients; renal perfusion has been shown to be worse in acute renal failure patients given dopamine alone [67]. On the other hand, combined low-dosages of dopamine/furosemide appear to be more useful due to improved renal function and potassium homeostasis (DAD-HF [68]). Promising results with regard to glomerular filtration have also been shown with the calcium sensitizing phosphodiesterase inhibitor, levosimendan (SURVIVE [69]), but its overall usefulness in patients with acute heart failure remains to be established and questions persist regarding their ability to improve kidney function and long-term survival. Vasopressin antagonists reduce symptoms in patients with hyponatremia and oliguria (EVEREST [70]) and adenosine receptor blockers improve kidney function. On the other hand, several clinical trials (PROTECT [71], REACH-UP [72]) report no functional benefit.

Angiotensin converting enzyme inhibitors could also improve clinical outcomes (CONSENSUS [73]) but it is recommended that they be used cautiously. Mineralocorticoid receptor blockers that markedly improve clinical outcomes in heart failure patients (RALES [74], EPHESUS [75]) are contra-indicated when renal dysfunction is present due to the elevated risk of hyperkalemia. Statins appear to reduce all-cause mortality in mild to moderate chronic kidney disease patients but may be ineffective in end-stage renal disease [76]. Reduced albuminuria that has been reported in rodents [77, 78] treated with statins affords significant renal protection; however, clinical findings on this question remain divided [79–81].

Non-pharmacologic treatments currently under investigation in clinical and animal studies also include renal denervation and remote conditioning. Renal denervation has been studied over the past 25 years to counter the effects of elevated renal sympathetic activity which 1-stimulates beta-1 receptors in the juxtaglomerular apparatus to increase renin release, 2- acts on alpha-1_B_ receptors of the collecting ducts to increase sodium reabsorption and 3- acts on alpha-1_A_ receptors of renal vasculature to promote vasoconstriction [82].

Renal *afferent* nerves transmit sensory information to the central nervous system from chemo- and mechanoreceptors in the kidneys; activation of renal afferent nerves is sympathoinhibitory in normal animals. Renal *efferent* nerves mediate changes in kidney function via innervation of all essential renal structures (renal vessels, tubules, juxtaglomerular apparatus); [82] activation of these nerves results in water retention, sodium reabsorption, reduced blood flow and activation of the renin-angiotensin-aldosterone system. Sympathoexcitatory reflexes are dominant in patients with resistant hypertension and CKD but the mechanisms involved in renal afferent activation are yet to be established. Renal nerves could play a role in renal inflammation and injury; suggested mechanisms include β-adrenergic receptor activation (causing podocyte injury), release of neuropeptides (neuropeptide Y, vasoactive intestinal polypeptide, substance P, etc.) that contribute to neuroimmune interactions, renin release from juxtaglomerular cells (increases plasma angiotensin II levels) and other pro-inflammatory cytokines including tumor necrosis factor and IL-1β from immune cells. Renal denervation has also been postulated to reduce kidney injury by improving blood glucose levels [83]. Findings from animal studies indicate that renal denervation mitigates inflammation and kidney injury; [84] however the results of clinical trials are inconsistent.

Two clinical trials, Simplicity HTN-1[85] and HTN-2 [86], report sustained reduction of blood pressure in patients with resistant hypertension and preserved renal function; [87] however, these beneficial effects were not observed in patients from the Simplicity HTN-3 trial [88]. Variability between studies with regard to renoprotection by renal denervation is probably related to differences in study design and primary endpoints. However, interventions on renal nerves merit further investigation for clinical management of hypertension and heart failure (i.e. major components of type 4 CRS phenotype). Interestingly, renal denervation may not be applicable in patients with AKI; numerous questions remain unanswered regarding the role of renal nerves in progression of acute renocardiac syndrome.

Remote conditioning stratagems (per-, pre- and post-conditioning) are the subject of a number of ongoing clinical trials (cf. ClinicalTrials.gov); numerous animal investigations have reported significant protection of vulnerable organs against ischemic injury by remote conditioning [89–93]. Ischemic tolerance of organs such as the heart may be reduced in the presence of co-morbidities; [32, 33, 94] larger myocardial infarcts occur in patients with chronic kidney disease and may account for higher mortality. In uremic rodents subject to different conditioning protocols significant protection against ischemic injury was observed; [95] similarly in a porcine model of AKI remote conditioning afforded marked renoprotective effects [96]. Remote limb-ischemia has also been shown to alleviate contrast medium induced renal injury in patients with moderate kidney disease [97] and even improved kidney function when performed prior to kidney transplantation in patients [98]. Whether organ conditioning stratagems could be useful in patients with acute renocardiac syndrome warrants further investigation; [99] patients with moderate chronic kidney disease have been reported to respond to remote conditioning [100]. These positive findings may be critical to improve outcomes in patients subject to renal replacement therapies. Ongoing clinical trials (The Context trial, clinicaltrials.gov NCT01395719, and the REPAIR Trial, ISRCTN30083294) should provide additional data regarding the potential usefulness of conditioning strategems for patients with renocardiac syndrome.

## Conclusions

Bi-directional communication between the heart and kidneys occurs through various pathways that, in healthy subjects, modulate cardiac output, vessel tone, volume status and excretion of metabolic waste compounds. It is increasingly apparent that pathological changes in one organ can instigate the release of a cascade of mediators that promote secondary dysfunction or injury in another distant organ. Further controlled clinical and more robust fundamental research is necessary to clarify existing contradictory findings and to improve understanding of mechanisms responsible for development of inter-organ pathologies.

## References

[1] Damman K, Testani JM. The kidney in heart failure: an update. Eur Heart J. 2015;36(23):1437-1444. 10.1093/eurheartj/ehv010 PMC4465636 25838436

[2] Maxwell MH, Breed ES, Schwartz IL (1950). Renal venous pressure in chronic congestive heart failure. J Clin Invest.

[3] Heywood JT (2004). The cardiorenal syndrome: lessons from the ADHERE database and treatment options. Heart Fail Rev.

[4] Shlipak MG, Massie BM (2004). The clinical challenge of cardiorenal syndrome. Circulation.

[5] Braam B, Joles JA, Danishwar AH, Gaillard CA (2014). Cardiorenal syndrome--current understanding and future perspectives. Nat Rev Nephrol.

[6] Ronco C, McCullough P, Anker SD, Anand I, Aspromonte N, Bagshaw SM, Bellomo R, Berl T, Bobek I, Cruz DN, Daliento L, Davenport A, Haapio M, Hillege H, House AA, Katz N, Maisel A, Mankad S, Zanco P, Mebazaa A, Palazzuoli A, Ronco F, Shaw A, Sheinfeld G, Soni S, Vescovo G, Zamperetti N, Ponikowski P (2010). Cardio-renal syndromes: report from the consensus conference of the acute dialysis quality initiative. Eur Heart J.

[7] Bonventre JV, Jorres A, Ronco C, Kellum JA (2010). Mechanisms of acute kidney injury and repair. Management of Acute Kidney Problems.

[8] Chuasuwan A, Kellum JA (2012). Cardio-renal syndrome type 3: epidemiology, pathophysiology, and treatment. Semin Nephrol.

[9] Joannidis M, Metnitz PG (2005). Epidemiology and natural history of acute renal failure in the ICU. Crit Care Clin.

[10] Bagshaw SM, Cruz DN, Aspromonte N, Daliento L, Ronco F, Sheinfeld G, Anker SD, Anand I, Bellomo R, Berl T, Bobek I, Davenport A, Haapio M, Hillege H, House A, Katz N, Maisel A, Mankad S, McCullough P, Mebazaa A, Palazzuoli A, Ponikowski P, Shaw A, Soni S, Vescovo G, Zamperetti N, Zanco P, Ronco C (2010). Epidemiology of cardio-renal syndromes: workgroup statements from the 7th ADQI Consensus Conference. Nephrol Dial Transplant.

[11] House AA, Anand I, Bellomo R, Cruz D, Bobek I, Anker SD, Aspromonte N, Bagshaw S, Berl T, Daliento L, Davenport A, Haapio M, Hillege H, McCullough P, Katz N, Maisel A, Mankad S, Zanco P, Mebazaa A, Palazzuoli A, Ronco F, Shaw A, Sheinfeld G, Soni S, Vescovo G, Zamperetti N, Ponikowski P, Ronco C (2010). Definition and classification of Cardio-Renal Syndromes: workgroup statements from the 7th ADQI Consensus Conference. Nephrol Dial Transplant.

[12] Cheung AK, Sarnak MJ, Yan G, Berkoben M, Heyka R, Kaufman A, Lewis J, Rocco M, Toto R, Windus D, Ornt D, Levey AS (2004). Cardiac diseases in maintenance hemodialysis patients: results of the HEMO Study. Kidney Int.

[13] Hebert K, Dias A, Delgado MC, Franco E, Tamariz L, Steen D, Trahan P, Major B, Arcement LM (2010). Epidemiology and survival of the five stages of chronic kidney disease in a systolic heart failure population. Eur J Heart Fail.

[14] de Abreu KL, Silva Junior GB, Barreto AG, Melo FM, Oliveira BB, Mota RM, Rocha NA, Silva SL, Araujo SM, Daher EF (2010). Acute kidney injury after trauma: Prevalence, clinical characteristics and RIFLE classification. Indian J Crit Care Med.

[15] Pavan M (2014). Incidence of acute cardiorenal syndrome type 3 in India. Iran J Kidney Dis.

[16] Liano F, Junco E, Pascual J, Madero R, Verde E (1998). The spectrum of acute renal failure in the intensive care unit compared with that seen in other settings. The Madrid Acute Renal Failure Study Group. Kidney Int Suppl.

[17] Varrier M, Ostermann M (2014). Novel risk factors for acute kidney injury. Curr Opin Nephrol Hypertens.

[18] Ryden L, Sartipy U, Evans M, Holzmann MJ (2014). Acute kidney injury after coronary artery bypass grafting and long-term risk of end-stage renal disease. Circulation.

[19] Ryden L, Ahnve S, Bell M, Hammar N, Ivert T, Sartipy U, Holzmann MJ (2014). Acute kidney injury after coronary artery bypass grafting and long-term risk of myocardial infarction and death. Int J Cardiol.

[20] Soto GJ, Frank AJ, Christiani DC, Gong MN (2012). Body mass index and acute kidney injury in the acute respiratory distress syndrome. Crit Care Med.

[21] Plataki M, Kashani K, Cabello-Garza J, Maldonado F, Kashyap R, Kor DJ, Gajic O, Cartin-Ceba R (2011). Predictors of acute kidney injury in septic shock patients: an observational cohort study. Clin J Am Soc Nephrol.

[22] Hsu RK, Hsu CY (2011). Proteinuria and reduced glomerular filtration rate as risk factors for acute kidney injury. Curr Opin Nephrol Hypertens.

[23] Grams ME, Astor BC, Bash LD, Matsushita K, Wang Y, Coresh J (2010). Albuminuria and estimated glomerular filtration rate independently associate with acute kidney injury. J Am Soc Nephrol.

[24] House AA (2012). Cardio-renal syndrome type 4: epidemiology, pathophysiology and treatment. Semin Nephrol.

[25] Shastri S, Sarnak MJ (2010). Cardiovascular disease and CKD: core curriculum 2010. Am J Kidney Dis.

[26] Tonelli M, Muntner P, Lloyd A, Manns BJ, Klarenbach S, Pannu N, James MT, Hemmelgarn BR (2012). Risk of coronary events in people with chronic kidney disease compared with those with diabetes: a population-level cohort study. Lancet.

[27] Washam JB, Herzog CA, Beitelshees AL, Cohen MG, Henry TD, Kapur NK, Mega JL, Menon V, Page RL, Newby LK (2015). Pharmacotherapy in chronic kidney disease patients presenting with acute coronary syndrome: a scientific statement from the American Heart Association. Circulation.

[28] Cruz DN, Gheorghiade M, Palazzuoli A, Ronco C, Bagshaw SM (2011). Epidemiology and outcome of the cardio-renal syndrome. Heart Fail Rev.

[29] Tonelli M, Wiebe N, Culleton B, House A, Rabbat C, Fok M, McAlister F, Garg AX (2006). Chronic kidney disease and mortality risk: a systematic review. J Am Soc Nephrol.

[30] Go AS, Chertow GM, Fan D, McCulloch CE, Hsu CY (2004). Chronic kidney disease and the risks of death, cardiovascular events, and hospitalization. N Engl J Med.

[31] Shroff GR, Frederick PD, Herzog CA (2012). Renal failure and acute myocardial infarction: clinical characteristics in patients with advanced chronic kidney disease, on dialysis, and without chronic kidney disease. A collaborative project of the United States Renal Data System/National Institutes of Health and the National Registry of Myocardial Infarction. Am Heart J.

[32] Wright RS, Reeder GS, Herzog CA, Albright RC, Williams BA, Dvorak DL, Miller WL, Murphy JG, Kopecky SL, Jaffe AS (2002). Acute myocardial infarction and renal dysfunction: a high-risk combination. Ann Intern Med.

[33] Shlipak MG, Heidenreich PA, Noguchi H, Chertow GM, Browner WS, McClellan MB (2002). Association of renal insufficiency with treatment and outcomes after myocardial infarction in elderly patients. Ann Intern Med.

[34] Fox CS, Muntner P, Chen AY, Alexander KP, Roe MT, Cannon CP, Saucedo JF, Kontos MC, Wiviott SD (2010). Use of evidence-based therapies in short-term outcomes of ST-segment elevation myocardial infarction and non-ST-segment elevation myocardial infarction in patients with chronic kidney disease: a report from the National Cardiovascular Data Acute Coronary Treatment and Intervention Outcomes Network registry. Circulation.

[35] Kusaba T, Humphreys BD (2014). Controversies on the origin of proliferating epithelial cells after kidney injury. Pediatr Nephrol.

[36] Lin F, Moran A, Igarashi P (2005). Intrarenal cells, not bone marrow-derived cells, are the major source for regeneration in postischemic kidney. J Clin Invest.

[37] Duffield JS, Bonventre JV (2005). Kidney tubular epithelium is restored without replacement with bone marrow-derived cells during repair after ischemic injury. Kidney Int.

[38] Romagnani P, Lasagni L, Remuzzi G (2013). Renal progenitors: an evolutionary conserved strategy for kidney regeneration. Nat Rev Nephrol.

[39] Guyton AC (1990). The surprising kidney-fluid mechanism for pressure control--its infinite gain!. Hypertension.

[40] Cannon PJ (1977). The kidney in heart failure. N Engl J Med.

[41] Stanton RC, Brenner BM (1986). Role of the kidney in congestive heart failure. Acta Med Scand Suppl.

[42] WINTON FR (1931). The influence of venous pressure on the isolated mammalian kidney. J Physiol.

[43] GOTTSCHALK CW, MYLLE M (1956). Micropuncture study of pressures in proximal tubules and peritubular capillaries of the rat kidney and their relation to ureteral and renal venous pressures. Am J Physiol.

[44] Deen WM, Robertson CR, Brenner BM (1972). A model of glomerular ultrafiltration in the rat. Am J Physiol.

[45] Damman K, van Deursen VM, Navis G, Voors AA, Van Veldhuisen DJ, Hillege HL (2009). Increased central venous pressure is associated with impaired renal function and mortality in a broad spectrum of patients with cardiovascular disease. J Am Coll Cardiol.

[46] Bongartz LG, Cramer MJ, Doevendans PA, Joles JA, Braam B (2005). The severe cardiorenal syndrome: 'Guyton revisited'. Eur Heart J.

[47] Sutton TA, Hato T, Mai E, Yoshimoto M, Kuehl S, Anderson M, Mang H, Plotkin Z, Chan RJ, Dagher PC (2013). p53 is renoprotective after ischemic kidney injury by reducing inflammation. J Am Soc Nephrol.

[48] Dagher PC, Mai EM, Hato T, Lee SY, Anderson MD, Karozos SC, Mang HE, Knipe NL, Plotkin Z, Sutton TA (2012). The p53 inhibitor pifithrin-alpha can stimulate fibrosis in a rat model of ischemic acute kidney injury. Am J Physiol Renal Physiol.

[49] Jackson G, Gibbs CR, Davies MK, Lip GY (2000). ABC of heart failure. Pathophysiology. BMJ.

[50] Ronco C, Haapio M, House AA, Anavekar N, Bellomo R (2008). Cardiorenal syndrome. J Am Coll Cardiol.

[51] Kingma JG, Simard D, Voisine P, Rouleau JR (2014). Impact of chronic kidney disease on myocardial blood flow regulation in dogs. Nephron Experimental Nephrology.

[52] Bidani AK, Griffin KA, Williamson G, Wang X, Loutzenhiser R (2009). Protective importance of the myogenic response in the renal circulation. Hypertension.

[53] Kingma JG, Vincent C, Rouleau JR, Kingma I (2006). Influence of acute renal failure on coronary vasoregulation in dogs. J Am Soc Nephrol.

[54] Wang Y, Bao X (2013). Effects of uric acid on endothelial dysfunction in early chronic kidney disease and its mechanisms. Eur J Med Res.

[55] Klassen PS, Lowrie EG, Reddan DN, DeLong ER, Coladonato JA, Szczech LA, Lazarus JM, Owen WF (2002). Association between pulse pressure and mortality in patients undergoing maintenance hemodialysis. J Am Med Assoc.

[56] Agarwal R (2009). Blood pressure components and the risk for end-stage renal disease and death in chronic kidney disease. Clin J Am Soc Nephrol.

[57] Muntner P, He J, Hamm L, Loria C, Whelton PK (2002). Renal insufficiency and subsequent death resulting from cardiovascular disease in the United States. J Am Soc Nephrol.

[58] Ejaz AA, Mohandas R (2014). Are diuretics harmful in the management of acute kidney injury?. Curr Opin Nephrol Hypertens.

[59] Ho KM, Sheridan DJ (2006). Meta-analysis of frusemide to prevent or treat acute renal failure. BMJ.

[60] Bagshaw SM, Delaney A, Haase M, Ghali WA, Bellomo R (2007). Loop diuretics in the management of acute renal failure: a systematic review and meta-analysis. Crit Care Resusc.

[61] Joannidis M, Druml W, Forni LG, Groeneveld AB, Honore P, Oudemans-van Straaten HM, Ronco C, Schetz MR, Woittiez AJ (2010). Prevention of acute kidney injury and protection of renal function in the intensive care unit. Expert opinion of the Working Group for Nephrology, ESICM. Intensive Care Med.

[62] KDIGO (2012). Clinical practice guideline for acute kidney injury. Kidney Int.

[63] Kovesdy CP, Trivedi BK, Kalantar-Zadeh K, Anderson JE (2006). Association of low blood pressure with increased mortality in patients with moderate to severe chronic kidney disease. Nephrol Dial Transplant.

[64] Sim JJ, Shi J, Kovesdy CP, Kalantar-Zadeh K, Jacobsen SJ (2014). Impact of achieved blood pressures on mortality risk and end-stage renal disease among a large, diverse hypertension population. J Am Coll Cardiol.

[65] Kim CS (2013). Pharmacologic Management of the Cardio-renal Syndrome. Electrolyte Blood Press.

[66] Klein L, Massie BM, Leimberger JD, O'connor CM, Pina IL, Adams KF, Califf RM, Gheorghiade M (2008). Admission or changes in renal function during hospitalization for worsening heart failure predict postdischarge survival: results from the Outcomes of a Prospective Trial of Intravenous Milrinone for Exacerbations of Chronic Heart Failure (OPTIME-CHF). Circ Heart Fail.

[67] Lauschke A, Teichgraber UK, Frei U, Eckardt KU (2006). 'Low-dose' dopamine worsens renal perfusion in patients with acute renal failure. Kidney Int.

[68] Giamouzis G, Butler J, Starling RC, Karayannis G, Nastas J, Parisis C, Rovithis D, Economou D, Savvatis K, Kirlidis T, Tsaknakis T, Skoularigis J, Westermann D, Tschope C, Triposkiadis F (2010). Impact of dopamine infusion on renal function in hospitalized heart failure patients: results of the Dopamine in Acute Decompensated Heart Failure (DAD-HF) Trial. J Card Fail.

[69] Mebazaa A, Nieminen MS, Packer M, Cohen-Solal A, Kleber FX, Pocock SJ, Thakkar R, Padley RJ, Poder P, Kivikko M (2007). Levosimendan vs dobutamine for patients with acute decompensated heart failure: the SURVIVE Randomized Trial. J Am Med Assoc.

[70] Konstam MA, Gheorghiade M, Burnett JC, Grinfeld L, Maggioni AP, Swedberg K, Udelson JE, Zannad F, Cook T, Ouyang J, Zimmer C, Orlandi C (2007). Effects of oral tolvaptan in patients hospitalized for worsening heart failure: the EVEREST Outcome Trial. J A M A.

[71] Voors AA, Dittrich HC, Massie BM, Delucca P, Mansoor GA, Metra M, Cotter G, Weatherley BD, Ponikowski P, Teerlink JR, Cleland JG, O'connor CM, Givertz MM (2011). Effects of the adenosine A1 receptor antagonist rolofylline on renal function in patients with acute heart failure and renal dysfunction: results from PROTECT (Placebo-Controlled Randomized Study of the Selective Adenosine A1 Receptor Antagonist Rolofylline for Patients Hospitalized with Acute Decompensated Heart Failure and Volume Overload to Assess Treatment Effect on Congestion and Renal Function). J Am Coll Cardiol.

[72] Gottlieb SS, Givertz MM, Metra M, Gergich K, Bird S, Jones-Burton C, Massie B, Cotter G, Ponikowski P, Weatherley B, O'Connor C, Dittrich H (2010). The effects of adenosine A(1) receptor antagonism in patients with acute decompensated heart failure and worsening renal function: the REACH UP study. J Card Fail.

[73] Ljungman S, Kjekshus J, Swedberg K (1992). Renal function in severe congestive heart failure during treatment with enalapril (the Cooperative North Scandinavian Enalapril Survival Study [CONSENSUS] Trial). Am J Cardiol.

[74] Pitt B, Zannad F, Remme WJ, Cody R, Castaigne A, Perez A, Palensky J, Wittes J (1999). The effect of spironolactone on morbidity and mortality in patients with severe heart failure. Randomized Aldactone Evaluation Study Investigators. N Engl J Med.

[75] Pitt B, Remme W, Zannad F, Neaton J, Martinez F, Roniker B, Bittman R, Hurley S, Kleiman J, Gatlin M (2003). Eplerenone, a selective aldosterone blocker, in patients with left ventricular dysfunction after myocardial infarction. N Engl J Med.

[76] Yagi S, Aihara K, Ikeda Y, Akaike M, Sata M, Matsumoto T (2012). Effects of statins on cardiorenal syndrome. Int J Vasc Med.

[77] Fujii M, Inoguchi T, Maeda Y, Sasaki S, Sawada F, Saito R, Kobayashi K, Sumimoto H, Takayanagi R (2007). Pitavastatin ameliorates albuminuria and renal mesangial expansion by downregulating NOX4 in db/db mice. Kidney Int.

[78] Liang XM, Otani H, Zhou Q, Tone Y, Fujii R, Mune M, Yukawa S, Akizawa T (2007). Renal protective effects of pitavastatin on spontaneously hypercholesterolaemic Imai Rats. Nephrol Dial Transplant.

[79] Strippoli GF, Navaneethan SD, Johnson DW, Perkovic V, Pellegrini F, Nicolucci A, Craig JC (2008). Effects of statins in patients with chronic kidney disease: meta-analysis and meta-regression of randomised controlled trials. BMJ.

[80] Atthobari J, Brantsma AH, Gansevoort RT, Visser ST, Asselbergs FW, Van Gilst WH, de Jong PE, de Jong-Van den Berg LT (2006). The effect of statins on urinary albumin excretion and glomerular filtration rate: results from both a randomized clinical trial and an observational cohort study. Nephrol Dial Transplant.

[81] Ruggenenti P, Perna A, Tonelli M, Loriga G, Motterlini N, Rubis N, Ledda F, Rota S, Satta A, Granata A, Battaglia G, Cambareri F, David S, Gaspari F, Stucchi N, Carminati S, Ene-Iordache B, Cravedi P, Remuzzi G (2010). Effects of add-on fluvastatin therapy in patients with chronic proteinuric nephropathy on dual renin-angiotensin system blockade: the ESPLANADE trial. Clin J Am Soc Nephrol.

[82] DiBona GF, Kopp UC (1997). Neural control of renal function. Physiol Rev.

[83] Mahfoud F, Schlaich M, Kindermann I, Ukena C, Cremers B, Brandt MC, Hoppe UC, Vonend O, Rump LC, Sobotka PA, Krum H, Esler M, Bohm M (2011). Effect of renal sympathetic denervation on glucose metabolism in patients with resistant hypertension: a pilot study. Circulation.

[84] Veelken R, Vogel EM, Hilgers K, Amann K, Hartner A, Sass G, Neuhuber W, Tiegs G (2008). Autonomic renal denervation ameliorates experimental glomerulonephritis. J Am Soc Nephrol.

[85] Simplicity HTN-1 Investigators (2011). Catheter-based renal sympathetic denervation for resistant hypertension: durability of blood pressure reduction out to 24 months. Hypertension.

[86] Esler MD, Krum H, Schlaich M, Schmieder RE, Bohm M, Sobotka PA (2012). Renal sympathetic denervation for treatment of drug-resistant hypertension: one-year results from the Symplicity HTN-2 randomized, controlled trial. Circulation.

[87] Veelken R, Schmieder RE (2014). Renal denervation--implications for chronic kidney disease. Nat Rev Nephrol.

[88] Bakris GL, Townsend RR, Liu M, Cohen SA, D'Agostino R, Flack JM, Kandzari DE, Katzen BT, Leon MB, Mauri L, Negoita M, O'Neill WW, Oparil S, Rocha-Singh K, Bhatt DL (2014). Impact of renal denervation on 24-hour ambulatory blood pressure: results from SYMPLICITY HTN-3. J Am Coll Cardiol.

[89] Kharbanda RK, Nielsen TT, Redington AN (2009). Translation of remote ischaemic preconditioning into clinical practice. Lancet.

[90] Przyklenk K, Bauer B, Ovize M, Kloner RA, Whittaker P (1993). Regional ischemic preconditioning protects remote virgin myocardium from subsequent coronary occlusion. Circulation.

[91] Vinten-Johansen J, Shi W (2011). Perconditioning and postconditioning: current knowledge, knowledge gaps, barriers to adoption, and future directions. J Cardiovasc Pharmacol Ther.

[92] Kingma JG (2014). Conditioning strategies limit cellular injury?. World Journal of Cardiovascular Diseases.

[93] Kingma JG, Simard D, Voisine P, Rouleau JR (2011). Role of the autonomic nervous system in cardioprotection by remote preconditioning in isoflurane-anaesthetized dogs. Cardiovasc Res.

[94] Dikow R, Kihm LP, Zeier M, Kapitza J, Tornig J, Amann K, Tiefenbacher C, Ritz E (2004). Increased infarct size in uremic rats: reduced ischemia tolerance?. J Am Soc Nephrol.

[95] Byrne CJ, McCafferty K, Kieswich J, Harwood S, Andrikopoulos P, Raftery M, Thiemermann C, Yaqoob MM (2012). Ischemic conditioning protects the uremic heart in a rodent model of myocardial infarction. Circulation.

[96] Gardner DS, Welham SJ, Dunford LJ, McCulloch TA, Hodi Z, Sleeman P, O'Sullivan S, Devonald MA (2014). Remote conditioning or erythropoietin before surgery primes kidneys to clear ischemia-reperfusion-damaged cells: a renoprotective mechanism?. Am J Physiol Renal Physiol.

[97] Igarashi G, Iino K, Watanabe H, Ito H (2013). Remote ischemic pre-conditioning alleviates contrast-induced acute kidney injury in patients with moderate chronic kidney disease. Circ J.

[98] Soendergaard P, Krogstrup NV, Secher NG, Ravlo K, Keller AK, Toennesen E, Bibby BM, Moldrup U, Ostraat EO, Pedersen M, Jorgensen TM, Leuvenink H, Norregaard R, Birn H, Marcussen N, Jespersen B (2012). Improved GFR and renal plasma perfusion following remote ischaemic conditioning in a porcine kidney transplantation model. Transpl Int.

[99] Crowley LE, McIntyre CW (2013). Remote ischaemic conditioning-therapeutic opportunities in renal medicine. Nat Rev Nephrol.

[100] Er F, Nia AM, Dopp H, Hellmich M, Dahlem KM, Caglayan E, Kubacki T, Benzing T, Erdmann E, Burst V, Gassanov N (2012). Ischemic preconditioning for prevention of contrast medium-induced nephropathy: randomized pilot RenPro Trial (Renal Protection Trial). Circulation.

